# Lung Deposition of a Radiolabeled Aerosol With Two Ventilation Modalities During Invasive Mechanical Ventilation: A Randomized Comparative Study

**DOI:** 10.1186/2197-425X-3-S1-A10

**Published:** 2015-10-01

**Authors:** J Dugernier, G Reychler, X Wittebole, J Roeseler, T Sottiaux, JB Michotte, R Vanbever, T Dugernier, P Goffette, MA Docquier, C Raftopoulos, P Hantson, F Jamar, PF Laterre

**Affiliations:** Université Catholique de Louvain, Cliniques Universitaires Saint Luc, Intensive Care Unit, Brussels, Belgium; Université Catholique de Louvain, Institut de Recherche Expérimentale et Clinique (IREC), Pneumology, Brussels, Belgium; Clinique Notre-Dame de Grâce, Intensive Care Unit, Gosselies, Belgium; Haute Ecole de Santé Vaud, Lausanne, Switzerland; Université Catholique de Louvain, Cliniques Universitaires Saint Luc, Louvain Drug Research Institute, Brussels, Belgium; Clinique Saint-Pierre, Intensive Care Unit, Ottignies, Belgium; Université Catholique de Louvain, Cliniques Universitaires Saint Luc, Department of Interventional Radiology, Brussels, Belgium; Université Catholique de Louvain, Cliniques Universitaires Saint Luc, Department of Anesthesiology, Brussels, Belgium; Université Catholique de Louvain, Cliniques Universitaires Saint Luc, Department of Neurosurgery, Brussels, Belgium; Université Catholique de Louvain, Cliniques Universitaires Saint Luc, Department of Nuclear Medicine, Brussels, Belgium

## Introduction

Volume-controlled ventilation has been suggested during nebulization to optimize lung deposition although promoting spontaneous ventilation is targeted for ventilated patient management. Comparing topographic lung aerosol deposition during volume-controlled and spontaneous ventilation in pressure support has never been performed.

## Objectives

The aim of this study was to compare lung deposition of a radiolabeled aerosol generated with a vibrating-mesh nebulizer during invasive mechanical ventilation, using two ventilation modes: pressure support ventilation (PS) and volume-controlled ventilation (VC).

## Methods

Seventeen postoperative neurosurgical patients without pulmonary disease volunteered to participate in the study and were randomly ventilated in PS (n = 8) or VC (n = 9) with constant inspiratory flow. Diethylenetriaminepentaacetic acid labelled with technetium-99 m (2 mCi/3 mL) was administered using a vibrating-mesh nebulizer (Aerogen Solo^®^, Aerogen Ltd., Galway, Ireland) connected to the endotracheal tube. Pulmonary and extrapulmonary particles deposition was analyzed by planar scintigraphy.

## Results

Mean lung deposition expressed as a percent of nominal dose was 10.5 ± 3.0% and 15.1 ± 5.0% during PS and VC, respectively (p < 0.05). Higher endotracheal tube and tracheal deposition was observed during PS (27.4 ± 6.6% versus 20.7 ± 6.0%, p < 0.05). a similar aerosol penetration from the inner to the outer region of the right lung (p = 0.347) and the left lung (p = 0.239) was observed.

## Conclusions

Volume-controlled ventilation improved lung deposition of aerosolized particles as compared to pressure support ventilation. The clinical benefit of this effect warrants further studies.

